# Biogenically synthesized gold nanocarrier ameliorated antiproliferative and apoptotic efficacy of doxorubicin against lung cancer

**DOI:** 10.3389/fphar.2024.1438237

**Published:** 2024-10-29

**Authors:** Yazeed Albulaihed, Prakriti Mishra, Mohd Saeed, Nadiyah M. Alabdallah, Tarig Ginawi, Irfan Ahmad Ansari

**Affiliations:** ^1^ Department of Biology, College of Science, University of Hail, Hail, Saudi Arabia; ^2^ Department of Biosciences, Integral University, Lucknow, India; ^3^ Department of Biology, College of Science, Imam Abdulrahman Bin Faisal University, Dammam, Saudi Arabia; ^4^ Basic and Applied Scientific Research Centre, Imam Abdulrahman Bin Faisal University, Dammam, Saudi Arabia; ^5^ Department of Biochemistry, College of Medicine, University of Hail, Hail, Saudi Arabia

**Keywords:** lung cancer, nanoformulation, gold nanoparticles, *Cannabis sativa L.*, doxorubicin, reactive oxygen species, mitochondrial membrane potential

## Abstract

**Introduction:**

Conventional chemotherapy treatment is commonly linked to significant side effects due to high therapeutic doses. In this regard, nanoformulations with chemotherapeutic medications hold promise in enhancing drug effectiveness through the reduction of therapeutic dosages, thereby mitigating the potential for adverse side effects. Because of numerous applications in the biomedical arena, there has been a rising interest in developing an environmentally acceptable, long-lasting, and affordable technique for the production of gold nanoparticles. In this particular context, the incorporation of plant extracts in the production of metallic nanoparticles has garnered the interest of numerous scholars. Here, we report the synthesis of gold particles by the green method using *Cannabis sativa L.* leaf extract and their conjugation with doxorubicin.

**Methods:**

The gold nanoparticles were synthesized by using *Cannabis sativa* extract and were characterized with various biophysical techniques. Subsequently, gold nanoparticles were conjugated with doxorubicin and their efficacy was tested on A549 cells.

**Results and Discussion:**

The biogenic synthesis of gold nanoparticles was ascertained through an absorption peak at a wavelength of 524 nm, and it was shifted to 527 nm when conjugated with doxorubicin. Nanoparticles were found to be stable exhibiting a zeta potential value of −20.1 mV, and it changed to −12.7 mV when loaded with doxorubicin. The hydrodynamic diameter of nanoparticles was determined to be 45.64 nm and it was increased to 58.95 nm when conjugated with the drug. The average size of nanoparticles analyzed by TEM was found to be approximately 17.2 nm, while it was 23.5 nm in the case of drug-nanoconjugate. Moreover, there was a significant amelioration in the antiproliferative potential of doxorubicin against lung cancer A549 cells when delivered with gold nanocarrier, which was evident by the lower IC50 and IC75 values of drug-nanoconjugates in comparison to drug alone. Furthermore, the inhibitory effect of drug-nanoconjugates and drug alone was characterized by alteration in the cell morphology, nuclear condensation, increased production of reactive oxygen species, abrogation of mitochondrial membrane potential, and enhanced caspase activities in A549 cells. In sum, our results suggested enhanced efficacy of doxorubicin-gold nanoconjugates, indicating effective delivery of doxorubicin inside the cell by gold nanoparticles.

## Introduction

Lung cancer is the second most frequently diagnosed cancer and is accountable for the highest number of cancer-related mortalities, with 22,00,000 new cancer cases and 18,00,000 fatalities in 2020 (Mogheri et al., 2021; Sung et al., 2021). In Saudia Arabia, lung carcinoma is among the foremost contributors to cancer-related mortality (Almatroudi, 2021). The incidence of lung cancer in Saudia Arabia has increased by a factor of 3.5 over the past 20 years (Almatroudi, 2021). Although in the advanced stage, first-line chemotherapy treatment involves platinum drugs as well as anthracyclines, taxanes, etc., with doxorubicin, being among the most effective. Nevertheless, low efficacy and substantial drug resistance along with severe side effects have been attributed to the use of these drugs Therefore, the development of new methods and approaches are prerequisite for the effective therapeutic management of lung cancer.

Novel nanoformulations of chemotherapeutic drugs could enhance their pharmacokinetic characteristics and thus, mitigate toxicity. Due to the significant side effects associated with traditional chemotherapeutic treatments such as doxorubicin, it is possible to decrease their dosage by combining them with a carrier or delivery agent. This approach aims to minimize the adverse effects of the drugs. Conjugating medications with a carrier facilitates targeted drug delivery to tumor cells, either nearby or directly within the cancer cells. Therefore, a reduced dosage of medication will yield greater efficacy compared to a higher therapeutic dosage. In this regard, gold nanoparticles (AuNPs) have been rigorously explored in the last 10 years due to their strong potential in the area of drug delivery, and chemotherapy (Aryal et al., 2009). AuNPs have drawn the most attention among nano-sized metal NPs due to their distinctive characteristics such as inertness, biocompatibility, and low toxicity (Anik et al., 2022). Adequately functionalized gold nanoparticles have the potential to act as a reservoir for drugs, in addition to offering extended circulation duration and minimal cytotoxicity (Aryal et al., 2009). The easy surface functionalization of AuNPs with different types of molecules makes them attractive candidates for delivering various payloads (Mukherjee et al., 2016). In this context, the utilization of green chemistry for the production of metal nanoparticles offers several advantages over traditional chemical techniques. Therefore, over the past decade, a significant endeavor has been undertaken to advance the field of green chemistry for the production of gold nanoparticles. Based on anecdotal and pre-clinical evidence, hemp or industrial hemp (Cannabaceae; *Cannabis sativa L.*) has been reported to possess numerous medicinal properties (Li et al., 2022). An array of metabolites such as terpenoids, flavonoids, and cannabinoids are present in the extracts of *Cannabis*. Delta-9-tetrahydrocannabinol (THC), a major cannabinoid of *Cannabis*, has been shown to exhibit many pharmacological attributes including analgesic, antioxidant, anti-inflammatory, antimicrobial, and anticancer properties (Lucas et al., 2018; MacCallum and Russo, 2018). Thus, we hypothesized that *C. sativa L.* extract could be a good choice for the synthesis and surface functionalization of AuNPs. *Cannabis sativa L.* extract has been used previously in a few studies to synthesize gold nanoparticles (Josiah et al., 2021; Singh et al., 2018a). Moreover, doxorubicin has also been encapsulated or attached with certain linkers with gold nanoparticles in some recent studies (Trejo-Teniente et al., 2024; Dhamecha et al., 2016; Khutale and Casey, 2017; Mirza and Shamshad, 2011; Wang et al., 2011). However, no report to date could show the conjugation of doxorubicin with gold nanoparticles, synthesized using *C. sativa L.* leaf extract. That’s why, the objective of the present investigation was to assess the potential of biogenically synthesized gold nanoparticles, using *C. sativa L.* extract, as a drug-carrying and delivery agent for the efficacious management of lung cancer.

## Materials and methods

### Materials

Doxorubicin, Tetrachloroauric (III) acid (HAuCl_4_.3H_2_O 99.9%), dyes like MTT, H_2_DCFDA, DAPI, and caspase colorimetric assay kits were purchased from Sigma. DMEM, FBS, Antibiotic-antimycotic solution, Pierce LDH Cytotoxicity Assay Kit, Mito Tracker Red CMXRos, and other cell culture reagents were procured from ThermoScientific, United States.

### Methods

#### Preparation of aqueous leaf extract of *Cannabis sativa L.*

*Cannabis sativa L.* plants were a kind gift from Dr. Maqbool Ahmad Khan, Deputy Director, Central Council for Research in Unani Medicine, Basha, Kursi Road, Lucknow, 226026, UP, India. Initially, *C. sativa L.* fresh leaves (10 g) were taken and subjected to two sequential washes with normal water followed by three subsequent washes with autoclaved Milli-Q water to ensure complete cleanliness. Further, the leaves were subjected to crushing to fine powder utilizing a mortar and pestle, followed by transfer to a 200 mL Erlenmeyer flask that was filled with 100 mL sterile Milli-Q water. The mixture was boiled for 5–10 min (Aljabali et al., 2018; Chouhan and Guleria, 2020). Finally, the mixture was cooled and supernatant was collected after centrifugation at 2000 rpm at 4°C for 10 min. The supernatant was used for the synthesis of the gold nanoparticles. The excess supernatant was stored at −20°C.

#### Stock of the gold salt solution

A solution of 1 mM HAuCl4 was prepared in autoclaved sterile Milli-Q water for the synthesis of the gold nanoparticles.

#### Synthesis of gold nanoparticles using the *Cannabis sativa L.* (CNB) leaf extract

In a typical 50 mL reaction, the green synthesis of the gold nanoparticles was undertaken by adding 5 mL of the CNB leaf extract to 45 mL of 1 mM aqueous HAuCl_4_ and subjected to incubation for 24–48 h at 37°C (Aljabali et al., 2018; Mukherjee et al., 2016). Reduction of Au^3+^ to Au^0^ was constantly monitored by recording the UV-Vis absorption spectrum as a function of time. Subsequently, biosynthesized gold nanoparticles (CNB-AuNPs) were purified by centrifugation at 14,000 r.p.m. at 4°C for 45 min. The intensely red loose pellet (CNB-AuNPs) was utilized for further characterization, as well as the *in vitro* experiments.

#### Characterization of gold nanoparticles (CNB-AuNPs)

The absorption of CNB-AuNPs was monitored by a UV-VIS spectrophotometer (Eppendorf Biospectrometer^®^ Kinetic, Eppendorf, United States). The size, shape, and morphology of the particles were determined by TEM (ThermoScientific™ Talos L120C, ThermoScientific, United States). Additionally, the hydrodynamic size and surface charge were measured using DLS (Zetasizer Nano-ZS, Model ZEN3600, Malvern Instrument Ltd., UK). To understand the role of possible functional groups, present in the plant extract, FTIR spectroscopy (Perkin Elmer Spectrum RX1, Perkin Elmer Inc., United States) was used.

#### Conjugation of doxorubicin (Dox) with CNB-AuNPs

Biosynthesized gold nanoparticles (CNB-AuNPs) were conjugated with doxorubicin by using 1-ethyl-3-(3-dimethylamino-propyl)-carbodiimide (EDC) as the activator (Timkovich, 1977). The 5 mL of reaction contained 5 mM EDC, 50 mM HEPES buffer, 250 µg CNB-AuNPs, and 250 µg doxorubicin. The mixture was incubated for 5 h at 37°C for the coupling reaction (Bagga et al., 2016; Iram et al., 2019). The drug-nanoconjugates (CNB-AuNPs-Dox) were purified by centrifugation at 14,000 r.p.m. at 4°C for 45 min and further used for the detailed characterization and *in vitro* experiments.

#### Quantification of Dox in CNB-AuNPs-Dox

A standard curve for Dox (1 μg/mL to 50 μg/mL) was prepared by plotting the absorbance of Dox at 481 nm versus the concentration of Dox in µg/mL. The concentration of Dox in the supernatant of CNB-AuNPs-Dox was measured by comparing its absorbance with the standard curve. From the standard curve, we calculated the % of Dox binding in CNB-AuNPs-Dox.

#### Cell culture

A549 cell line was purchased from the NCCS, Pune, India. The cells were cultured in DMEM medium, which was enriched with 10% FBS and 1% antibiotic-antimycotic solution. The cells were grown at 37°C in a humidified incubator with 5% CO_2_.

#### Cytotoxicity evaluation

To test the efficacy of CNB-AuNPs-Dox, A549 cells (1 × 10^4^/well) were grown in a 96-well plate and subjected to 24 h of incubation at 37°C. The cells were subjected to treatment with varying doses of CNB-AuNPs-Dox followed by an incubation period of 24 h (Mishra et al., 2022). Following incubation, the medium was removed from each well and 10 µL of MTT (5 mg/mL) was introduced to the respective wells. The plate was further kept at 37°C for 2 h. The crystals of formazan, that were produced, were solubilized in 100 µL of dimethyl sulfoxide. The absorbance of each well was taken at 570 nm with a reference filter of 630 nm using a microplate reader. The result was expressed as percent cell viability relative to control.

#### Lactate dehydrogenase (LDH) release assay

LDH release assay was also performed to assess CNB-AuNPs-Dox-mediated cytotoxicity in A549 cells. Briefly, cells (5 × 10^3^ per well) were placed in a 96-well plate, followed by co-culturing with CNB-AuNPs-Dox, for 24 h. Subsequently, LDH activity was measured in all treatment groups as per the manufacturer’s instruction. Later, % cytotoxicity among all treatment groups was calculated by the given formula:

$$\%\;Cytotoxicity=\frac{\left(Drug\;Treated\;LDH\;activity\right)-\left(Spontaneous\;LDH\;activity\right)}{Maximum\;LDH\;activity-Spontaneous\;LDH\;activity}\times 100$$

#### Assessing morphological changes in A549 cells

Morphological alterations in CNB-AuNPs-Dox-treated lung cancer cells were analyzed by phase contrast microscopy. Briefly, A549 cells (5 × 10^3^/well) were grown in a 96-well plate and subjected to 24 h incubation. Thereafter, cells were treated with IC_50_ and IC_75_ of CNB-AuNPs-Dox and incubated for 24 h at 37°C. Subsequently, the alterations in cell morphology of treated and control cells were examined using FLoid imaging station (ThermoScientific, United States).

#### Examination of nuclear alterations

The apoptotic effect of CNB-AuNPs-Dox was investigated against A549 cells using DAPI staining. The cells were grown and treated with nanoconjugates in the same manner as mentioned above. Subsequently, the cells were rinsed with PBS and fixed for 10 min in 4% paraformaldehyde. Later, the cells were permeabilized (3% paraformaldehyde and 0.5% Triton X-100) and stained with DAPI. Later, cells were observed under a blue filter (Excitation: Emission:390/40 nm: 446/33 nm) using FLoid Imaging station.

#### Mitochondrial membrane potential (∆Ψm) assessment

The ∆Ψm was measured in A549 cells, treated with CNB-AuNPs-Dox, using Mito Tracker Red CMX Ros labeling. In a 24-well plate, cells (1 × 10^5^ per well) were seeded and allowed for 24 h incubation. Cells were then co-cultured for 24 h with CNB-AuNPs-Dox. Further, cells were washed and stained for 30 min in the dark with Mito Tracker Red (300 nM), and images were captured using FLoid Imaging station.

#### Intracellular reactive oxygen species (ROS) generation

The fluorogenic dye H_2_DCFDA was used to detect A549 cells treated with CNB-AuNPs-Dox. A549 cells (1 × 10^4^/well) were seeded and subjected to overnight incubation at 37°C. Subsequently, cells were treated for another 24 h with varying doses of CNB-AuNPs-Dox, followed by staining with H_2_DCFDA (25 µM) for 30 min at 37°C. Later, in each well, the media was replaced with 200 µL of PBS for washing. Finally, images were captured using FLoid imaging station.

Additionally, to determine the amount of ROS, cells (1 × 10^4^/well) were grown a in 96-well black bottom culture plate and subjected to overnight incubation at 37°C. A549 cells were then exposed to CNB-AuNPs-Dox for another 12 h, followed by incubation with H_2_DCFDA (25 µM) for 30 min at 37°C. Fluorescence intensity was recorded via a multi-mode microplate reader.

#### Determination of caspase activity

Briefly, A549 cells were treated with different concentrations of CNB-AuNPs-Dox and incubated for another 24 h. Later, caspase activities were measured according to the manufacturer’s instructions.

#### Statistical analysis

Data shown in this study are mean ± standard deviation (S.D.) of three individual experiments performed in triplicate. Statistical analysis was done using one-way ANOVA followed by Dunnett post-hoc test and two-way ANOVA with Tukey multiple comparison test (*p < 0.05, **p < 0.01, and ***p < 0.001 denote level of significance between means of treatment groups).

## Results

### *Cannabis sativa L.* leaf extract mediated synthesis of gold nanoparticles (AuNPs)

At the outset, a sequence of experiments was executed to optimize the reaction conditions to biosynthesize gold nanoparticles (AuNPs). Subsequently, the results indicated that CNB leaf extract produced stable gold nanoparticles (CNB-AuNPs) which were used for the detailed characterization as well as the *in vitro* experiments. Secondary metabolites and reducing enzymes of the aqueous CNB leaf extract are thought to trigger the synthesis of gold nanoparticles (CNB-AuNPs).

### Characterization of CNB-AuNPs and conjugation of Dox with AuNPs

**FIGURE 1:**
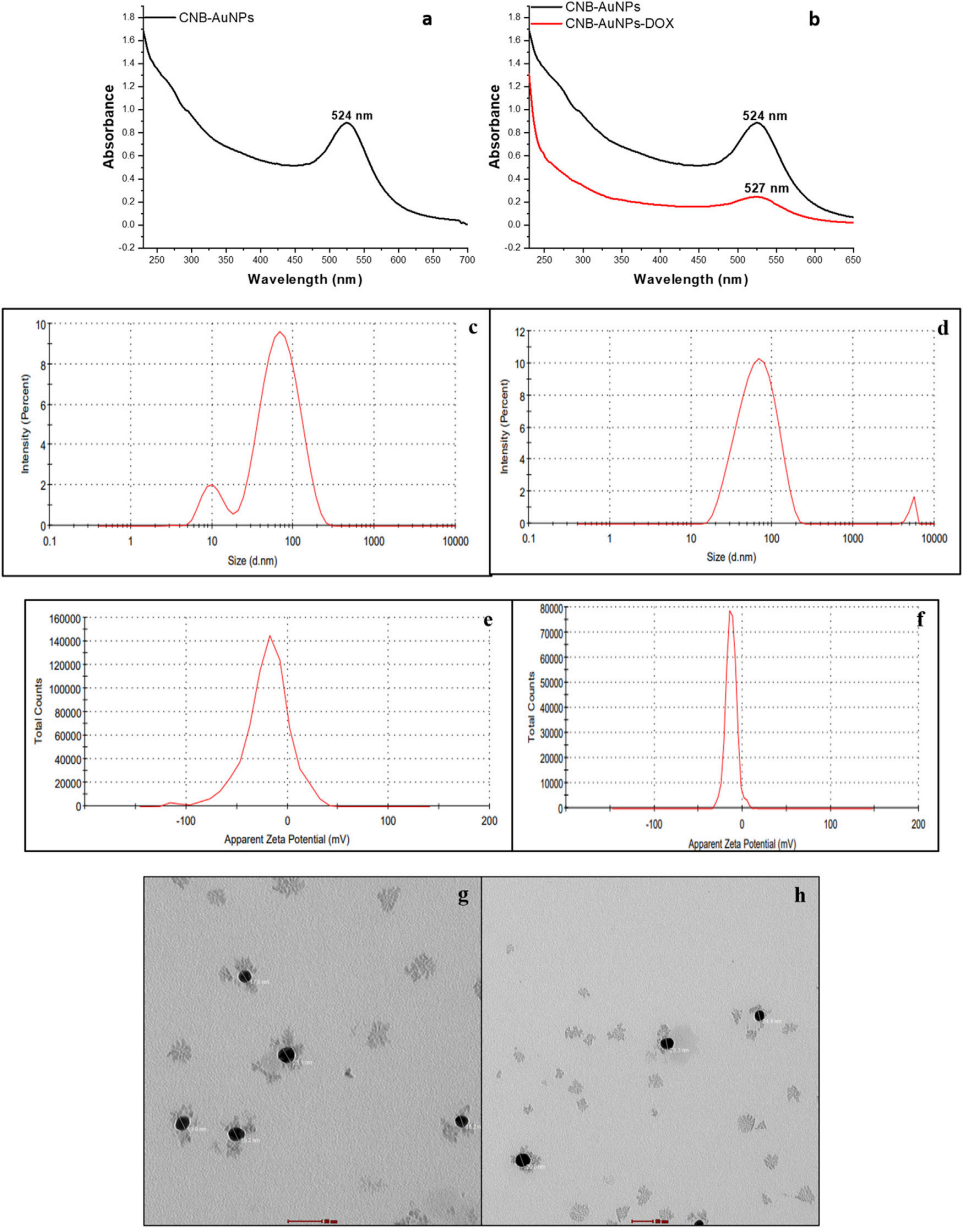
Characterization of CNB-AuNPs **(A, C, E, G)** and CNB-AuNPs-Dox **(B, D, F, H)** by UV-Visible spectra, Hydrodynamic diameter, Zeta potential, and Transmission Electron Microscopy, respectively.

**FIGURE 2:**
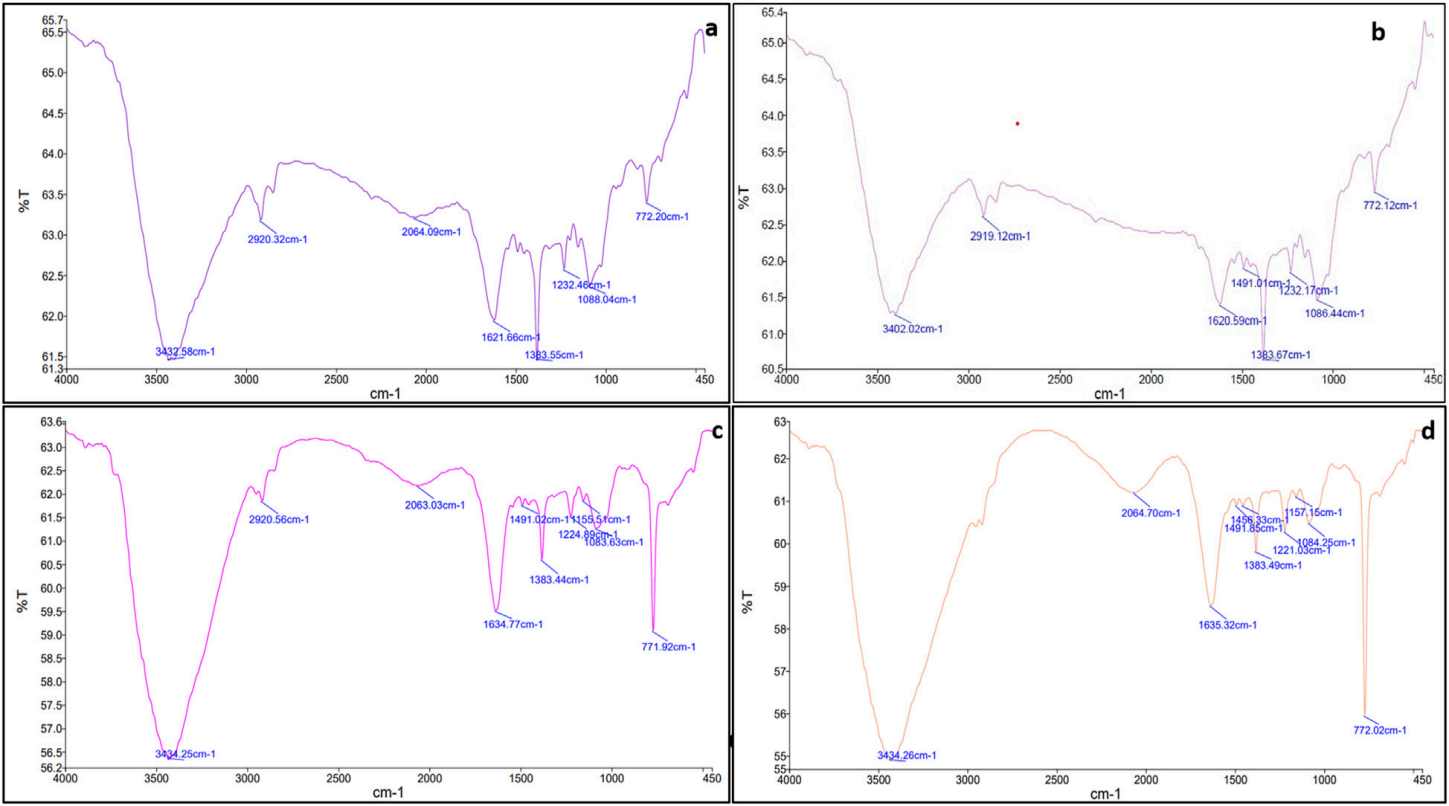
FTIR spectra of **(A)** *Cannabis sativ*a leaf extract **(B)** CNB-AuNPs **(C)** Dox **(D)** CNB-AuNPs-Dox.

Upon mixing the leaf extract with the aqueous solution of chloroauric acid (HAuCl4), the change in solution color from light yellow to ruby red indicated the synthesis of gold nanoparticles (CNB-AuNPs). An absorbance peak at 524 nm was observed corresponding to the characteristic surface plasmon resonance of gold (Figure 1A). The result has substantiated the synthesis of AuNPs, given that the existence of a peak at approximately 524 nm serves as a distinctive indication to recognize gold nanoparticles (Park et al., 2023). The dynamic light extracting (DLS) method was used to determine the average particle size and distribution of CNB-AuNPs, which had an average particle size of 45.64 nm as shown in Figure 1C. Furthermore, the zeta potential of the synthesized CNB-AuNPs was found to be −20.1 mV at room temperature, suggesting the high stability of nanoparticles (Figure 1E). The TEM micrograph confirmed the average size of CNB-AuNPs to be 17.2 nm and also, they seemed to be mono-dispersed (Figure 1G). The FTIR spectra showed that gold nanoparticles (Figure 2B) exhibited the chemical groups present in the leaf extract (Figure 2A). The FTIR spectrum of CNB-AuNPs exhibited medium absorption bands at 3,402.02 cm^−1^ due to O-H stretching vibrations of alcohols (Datkhile et al., 2023). The peak around 2,919.12 cm^−1^ corresponds to C-H stretching. The band at 1,620.59 cm^−1^ could be linked with a C=C conjugated bond (El-Deeb et al., 2022). The peak near 1,383.67 cm^−1^ and 1,086.44 cm^−1^ corresponds to vibration of the O-H (alcohol) and C-N (amines) stretch, respectively.

Conjugation of Dox with AuNPs was characterized by a shift in the spectra towards a higher wavelength (from 524 nm to 527 nm) as observed by UV-Vis spectroscopy (Figure 1B). Further, the hydrodynamic diameter of particles was found to be increased to 58.95 nm after conjugation (Figure 1D). The zeta potential of CNB-AuNPs-Dox was found to be −12.7 mV (Figure 1F). TEM depicts the image of CNB-AuNPs-Dox with an increase in average size (23.5 nm) which further confirmed conjugation. After conjugation, it was observed that the mean size had increased marginally in comparison to that of AuNPs, owing to the attachment of Dox onto the surface of the nanoparticles. CNB-AuNPs-Dox nanoconjugates were observed to possess a spherical shape and exhibit mono-dispersed characteristics, as confirmed by the TEM micrograph (Figure 1H). The FTIR spectra confirmed the conjugation of Dox with gold nanoparticles (Figures 2C, D). In the FTIR spectrum of Dox, the bend peaks of N-H bonds were observed at 1,634.77 cm^−1^ and N-H stretching at 3,434.25 cm^−1^. The bend peaks of O-H bonds (phenol) were also observed at 1,383.44 cm^−1^. Two peaks of C-N (amine) stretching at 1,155.51 cm^−1^ and 1,083.63 cm^−1^ were also observed. The spectrum of CNB-AuNPs-Dox was compared with Dox and it was found that the medium absorption band at 3,434.26 cm^−1^ was due to N-H stretching vibrations of amide bond present on AuNPs confirming conjugation of Dox. Further, the peak at 1,635.32 cm^−1^ probably represents the N-H group bending of the amine group of Dox. The bend peaks of O-H bonds (phenol) were also shifted and observed at 1,383.49 cm^−1^. A peak at 1,084.25 cm^−1^ corresponds to a stretch of C-N of peptide bond which also confirmed the conjugation of Dox.

### Drug loading efficiency

After conjugation, the quantitative estimation of the loading of Dox on CNB-AuNPs was carried out by taking the absorbance of both the total drug added and unbound drug following conjugation via UV-Vis spectroscopy at a wavelength of 481 nm. The amount of Dox conjugated to CNB-AuNPs was found to be 46.24% ± 3.98%, thus revealing substantial binding of the drug with nanoparticles.

### *In vitro* cytotoxicity of gold-drug nanoconjugates

**FIGURE 3:**
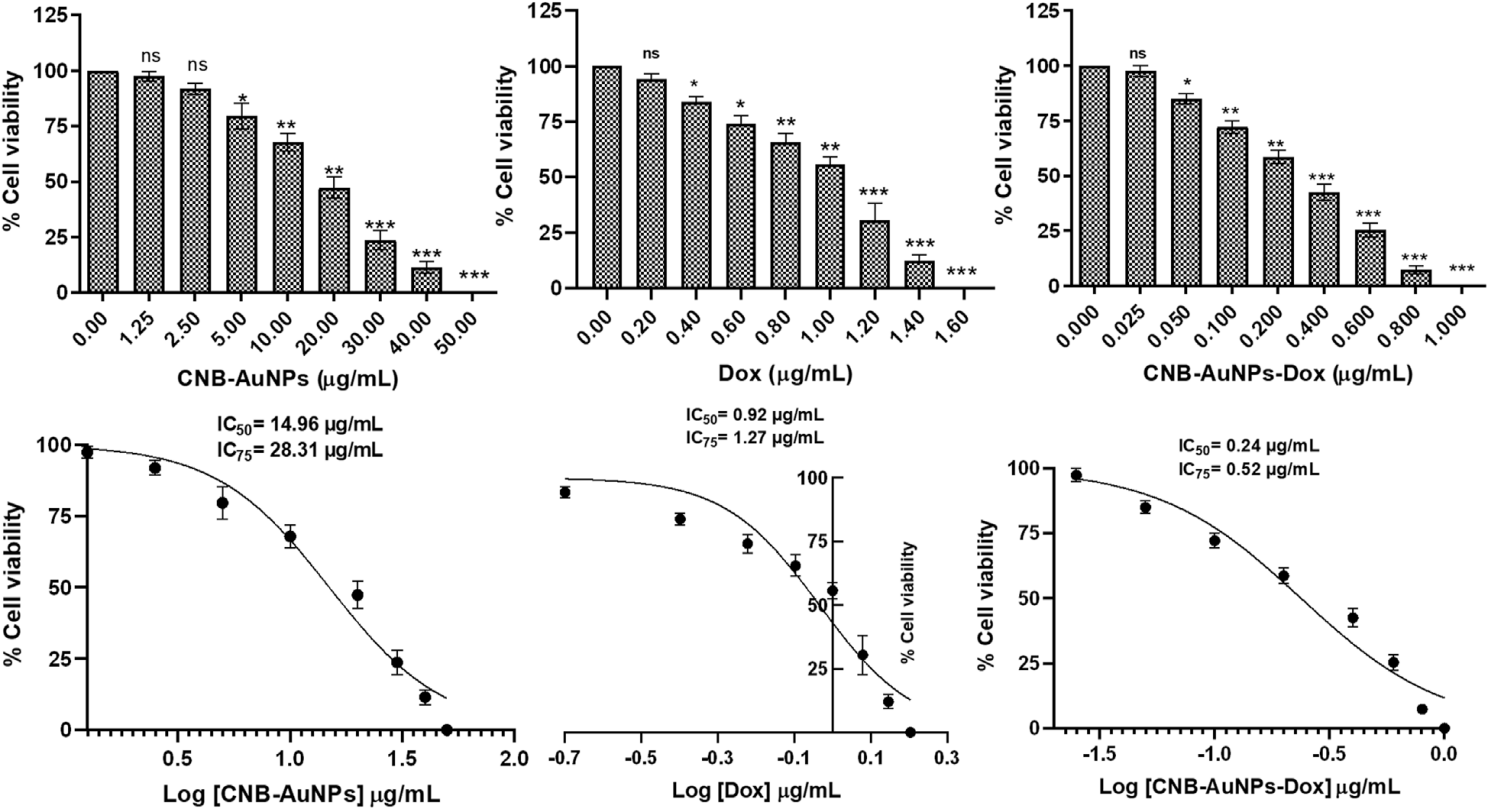
Percent cell viability of A549 cells of CNB-AuNPs-Dox, Dox, and CNB-AuNPs for 24 h assessed by MTT assay along with their respective IC_50_ and IC_75_ values. The results presented are the mean ± SD of three independent experiments performed in triplicate. Significance among different dosage groups was determined using one-way ANOVA followed by the Dunnett post-hoc test (where *p < 0.05, **p < 0.01, and ***p < 0.001 represent significant differences compared with vehicle control).

To assess the responsiveness of lung carcinoma cells to drug-loaded nanoparticles and establish their optimal dosage, A549 cells were subjected to varying concentrations of drug-nanoconjugates for a duration of 24 h, following which the percentage of cell viability was calculated by performing MTT assay. As can be seen in Figure 3, CNB-AuNPs-Dox (0.025–0.80 μg/mL) substantially inhibited the viability of A549 cells after 24 h of treatment. At the dose of 0.025, 0.05, 0.1, 0.2, 0.4, 0.6, and 0.80 μg/mL, the cell viability was found to be 97.40% ± 1.47%, 84.94% ± 1.39%, 72.14% ± 1.66%, 58.69% ± 1.75%, 42.55% ± 2.08%, 25.41% ± 1.81%, and 7.36% ± 1.01%, respectively (Figure 3). Similarly, Dox also reduced the survival of A549 cells at the dose of 0.2, 0.4, 0.6, 0.8 1.0, 1.2, and 1.4 μg/mL; and viability was found to be 94.26% ± 2.29%, 83.80% ± 2.38%, 74.12% ± 3.60%, 65.56% ± 4.22%, 55.69% ± 3.27%, 30.44% ± 7.69%, and 12.08% ± 2.75%, respectively (Figure 3). Furthermore, CNB-AuNPs alone, at the dose of 0.2, 0.4, 0.6, 0.80. and 1.0 μg/mL, exerted insignificant effect on A549 cells (Figure 3). On analyzing the data, we observed that gold-doxorubicin nanoconjugates induce a strong cytotoxic effect in comparison to doxorubicin at similar doses of treatment, while CNB-AuNPs alone exhibited an insignificant effect on A549 cells at equivalent concentrations. However, CNB-AuNPs were found to be cytotoxic against A549 cells in the range of 5–50 μg/mL (Figure 3).

The IC_50_ and IC_75_ of CNB-AuNPs-Dox were found to be 0.24 μg/mL and 0.52 μg/mL, respectively. Similarly, IC_50_ and IC_75_ of Dox were 0.92 μg/mL and 1.27 μg/mL, respectively. However, IC_50_ and IC_75_ of CNB-AuNPs alone were 14.96 μg/mL and 28.31 μg/mL, respectively. Here, the isoeffective doses of CNB-AuNPs-Dox were substantially reduced in comparison to Dox alone which signified that CNB-AuNPs-Dox exert a similar effect on A549 cells at a much lower dose in comparison to Dox. Thus, the result suggested the effective delivery of doxorubicin inside cells by drug-gold nanoconjugates (Figure 5).

### LDH release assay

**FIGURE 4:**
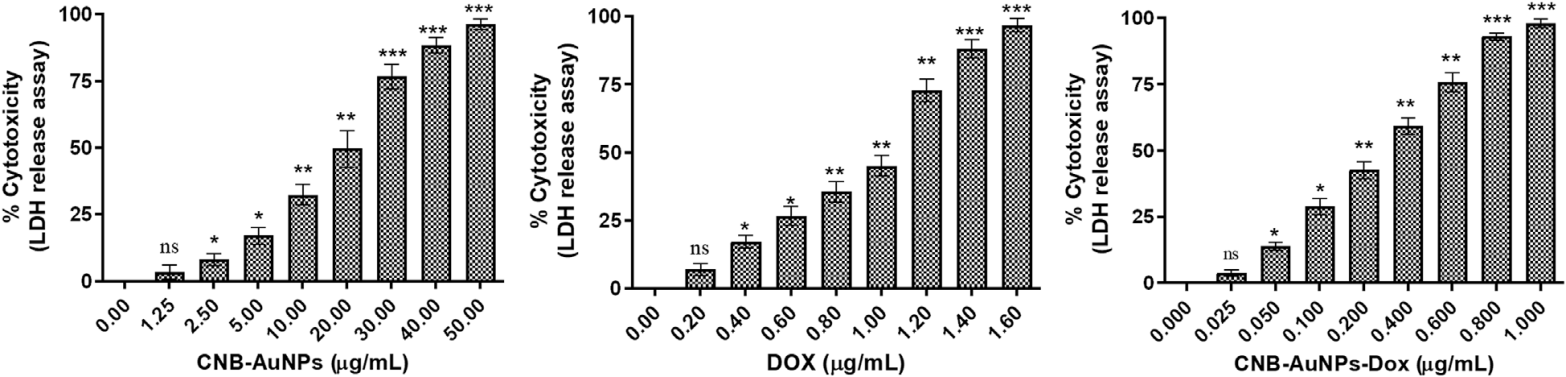
Percent cytotoxicity of A549 cells treated with various doses of CNB-AuNPs-Dox, Dox, and CNB-AuNPs for 24 h assessed by LDH release assay. The results presented are the mean ± SD of three independent experiments performed in triplicate. Significance among different dosage groups was determined using one-way ANOVA followed by the Dunnett post-hoc test (where *p < 0.05, **p < 0.01, and ***p < 0.001 represent significant differences compared with vehicle control).

**FIGURE 5:**
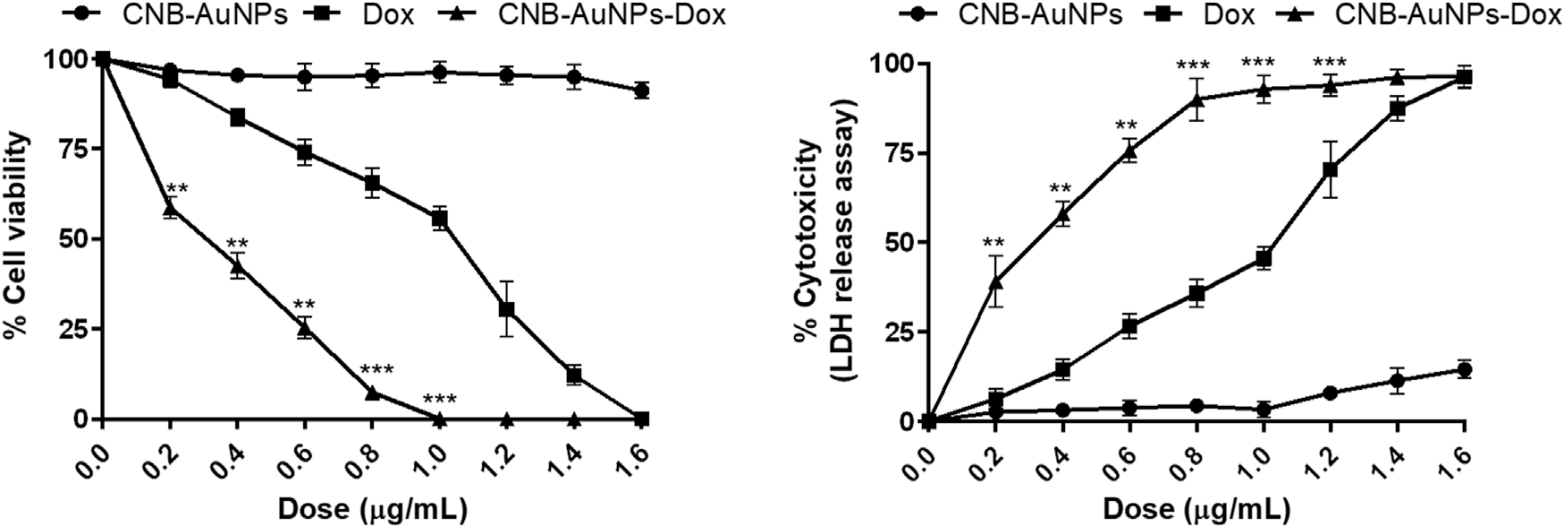
Comparison of the efficacy of CNB-AuNPs-Dox, Dox, and CNB-AuNPs against A549 cells in terms of cell viability and cytotoxicity. The results presented are the mean ± SD of three independent experiments performed in triplicate. Significance among different dosage groups was determined using two-way ANOVA followed by Tukey’s multiple comparison tests (where *p < 0.05, **p < 0.01, and ***p < 0.001 represent significant differences between means of different treatment groups).

The results showed that treatment of CNB-AuNPs-Dox for 24 h, caused substantial cell death in A549 cells, as compared to untreated control; and percent cytotoxicity was found to be 3.76% ± 0.73%, 13.97% ± 0.83%, 28.88% ± 1.79%, 42.62% ± 1.85%, 59.12% ± 1.74%, 75.71% ± 2.09%, and 92.93% ± 0.79% at the dose of 0.025, 0.05, 0.1, 0.2, 0.4, 0.6 and 0.80 μg/mL, respectively (Figure) Similarly, Dox also caused cytotoxicity of 6.97% ± 1.24%, 17.16% ± 1.37%, 26.55% ± 2.04%, 35.51% ± 2.25%, 45.05% ± 2.14%, 72.84% ± 2.44%, and 88.11% ± 1.92% at the dose of 0.2, 0.4, 0.6, 0.80, and 1.0 μg/mL, respectively (Figure). Additionally, CNB-AuNPs alone exerted an insignificant effect on A549 cells at the dose of 0.2, 0.4, 0.6, 0.80, and 1.0 μg/mL (Figure 4). Thus, the results showed that drug-gold nanoconjugate caused more cytotoxicity in A549 cells at similar doses when compared to Dox alone, which could be due to efficient uptake of drug by cells via drug-gold nanocarrier (Figure 5).

### Cytomorphological changes in A549 cells

**FIGURE 6:**
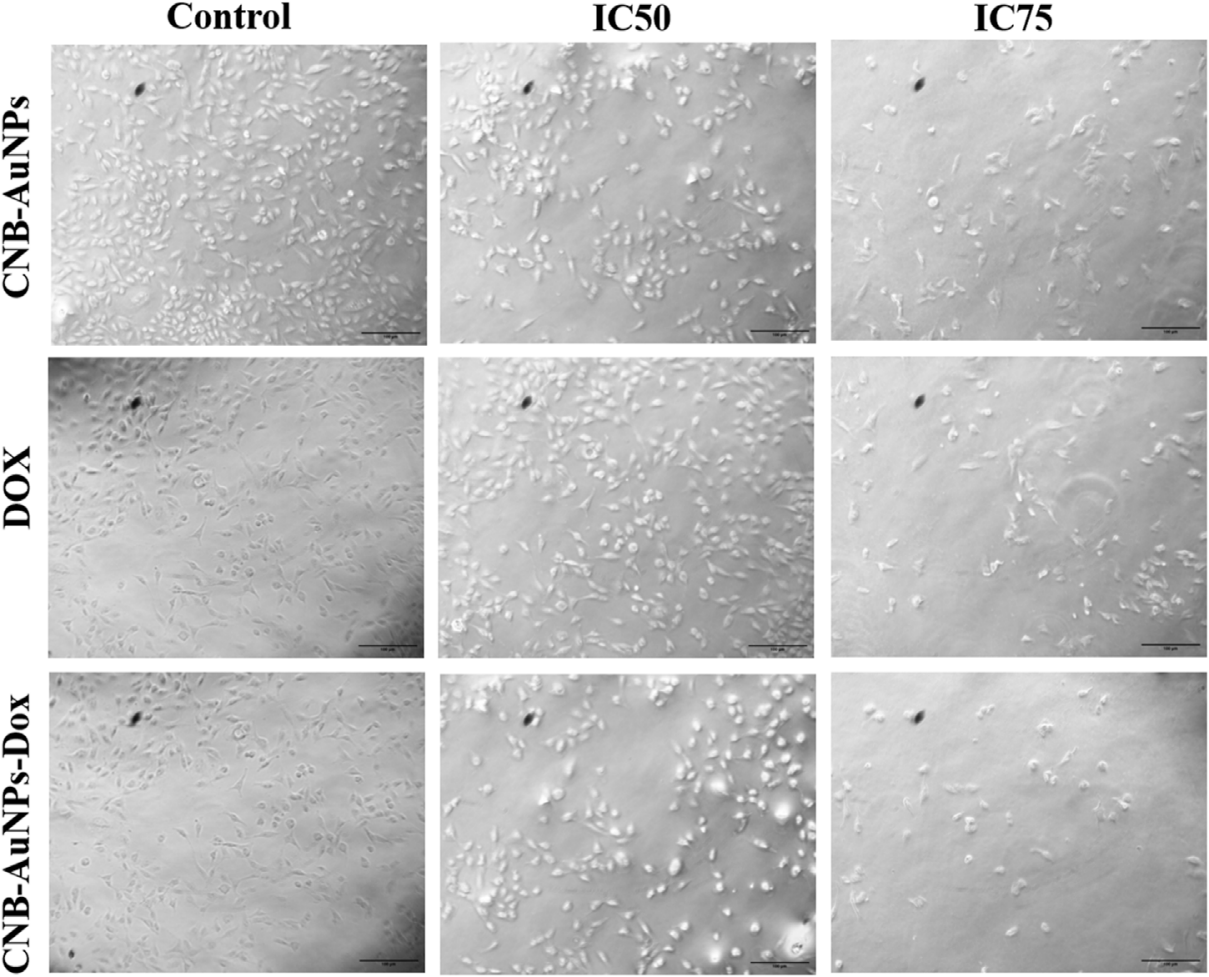
Cytomorphological images of A549 cells treated with IC50 and IC75 of CNB-AuNPs-Dox, Dox, and CNB-AuNPs for 24 h were analyzed by phase contrast microscopy. The images shown are representative of three independent experiments performed in triplicate (Magnification ×20; Scale bar 100 µm).

To analyze cytomorphological changes in A549 cells, caused by drug-gold nanoconjugates, cells were co-cultured with IC_50_ and IC_75_ of CNB-AuNPs-Dox as well as Dox and CNB-AuNPs and were observed under a microscope. A549 cells in all the treated groups exhibited a change in cell morphology that was dependent on the dose administered (Figure 6). The cells in the untreated control group exhibited a well-spread, flattened morphology. Conversely, the A549 cells in all treatment groups displayed a rounded morphology with slight shrinkage. A subset of cells exhibited signs of cytotoxicity in A549 cells, as evidenced by cellular swelling, and cell membrane lysis. As the treatment dose was increased from IC_50_ to IC_75_, these changes in A549 cells were substantially increased in all the treatment groups. Here also, CNB-AuNPs-Dox instigated comparable changes in A549 cells at lesser doses in comparison to DOX, which was in line with our above results.

### Nuclear changes in A549 cells

**FIGURE 7:**
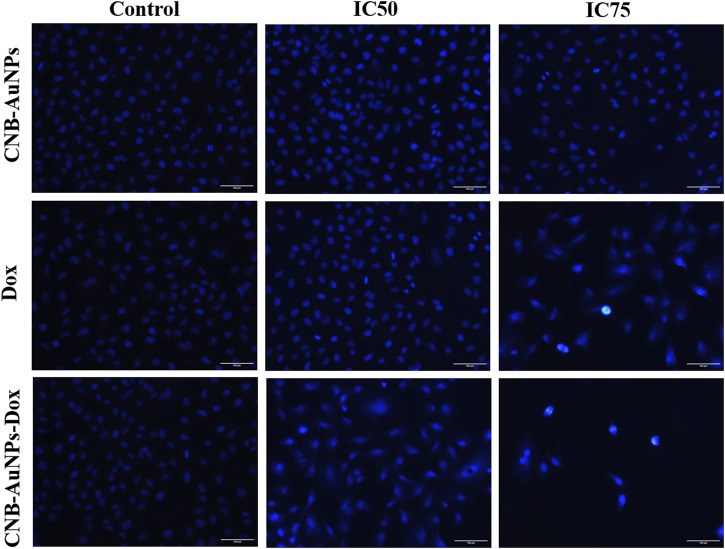
Nuclear morphology of DAPI stained nuclei of A549 cells treated with IC50 and IC75 of CNB-AuNPs-Dox, Dox, and CNB-AuNPs for 24 h analyzed by fluorescence microscopy. The images shown are representative of three independent experiments (Scale bar: 100μm; Magnification: ×20).

DAPI staining was conducted to probe and determine if the suppression of cell proliferation in A549 cells treated with CNB-AuNPs-Dox was indeed a result of apoptosis. As shown in Figure 7, significant nuclear alterations were observed in cells among all the treatment groups (Figure 7). CNB-AuNPs-Dox, Dox, and CNB-AuNPs, at their respective IC_50_ and IC_75_, induced nuclear changes in A549 cells, whereas the cells showed normal nuclei in the control group. As the dose was increased from IC_50_ to IC_75_, the nuclear changes in A549 cells were significantly increased in all the treated groups. As observed above, CNB-AuNPs-Dox exerted corresponding nuclear alterations in A549 cells at lower doses when compared with Dox.

### Reduction in the mitochondrial membrane potential (ΔΨm)

**FIGURE 8:**
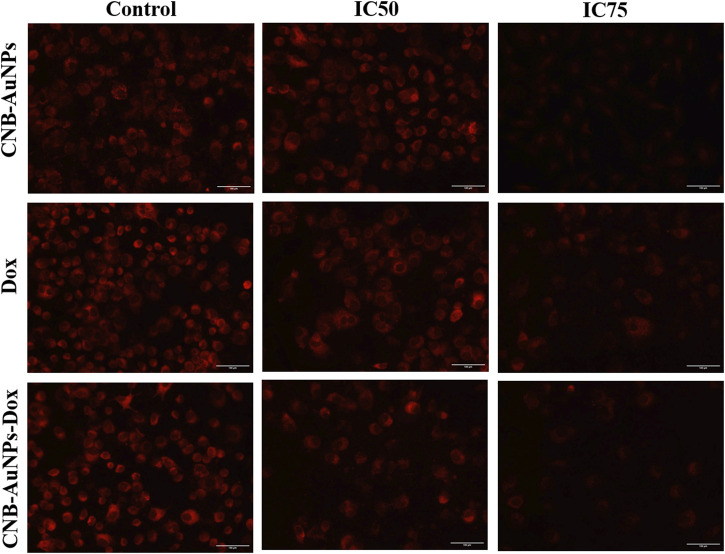
Qualitative assessment of Mitochondrial Membrane Potential in Mitotracker Red-stained A549 cell treated with IC50 and IC75 of CNB-AuNPs-Dox, Dox, and CNB-AuNPs for 24 h analyzed by fluorescence microscopy. The images shown are representative of three independent experiments (Scale bar: 100 μm; Magnification: ×20).

The mitochondrial pathway of apoptosis is activated by the abrogation of the ΔΨm. Thus, to determine, whether apoptosis induced in CNB-AuNPs-Dox-treated lung cancer cells was due to disruption of ΔΨm, A549 cells were co-cultured with IC_50_ and IC_75_ of CNB-AuNPs-Dox as well as Dox and CNB-AuNPs for 24 h and were examined after staining with Mitotracker Red CMXRos dye. Subsequently, a substantial reduction in ΔΨm of A549 cells was observed in all the treated groups (Figure 8). The maximum fluorescence intensity of the Mitotracker dye was seen in untreated control cells followed by a gradual decline in the fluorescence among cells of all the treated groups. As the dose was increased from IC_50_ to IC_75_, ΔΨm in A549 cells was further decreased in all the treatment groups. Like previous observations, CNB-AuNPs-Dox caused an equivalent reduction in ΔΨm in A549 cells at lower doses when compared with Dox alone.

### Augmentation of reactive oxygen species (ROS)

**FIGURE 9:**
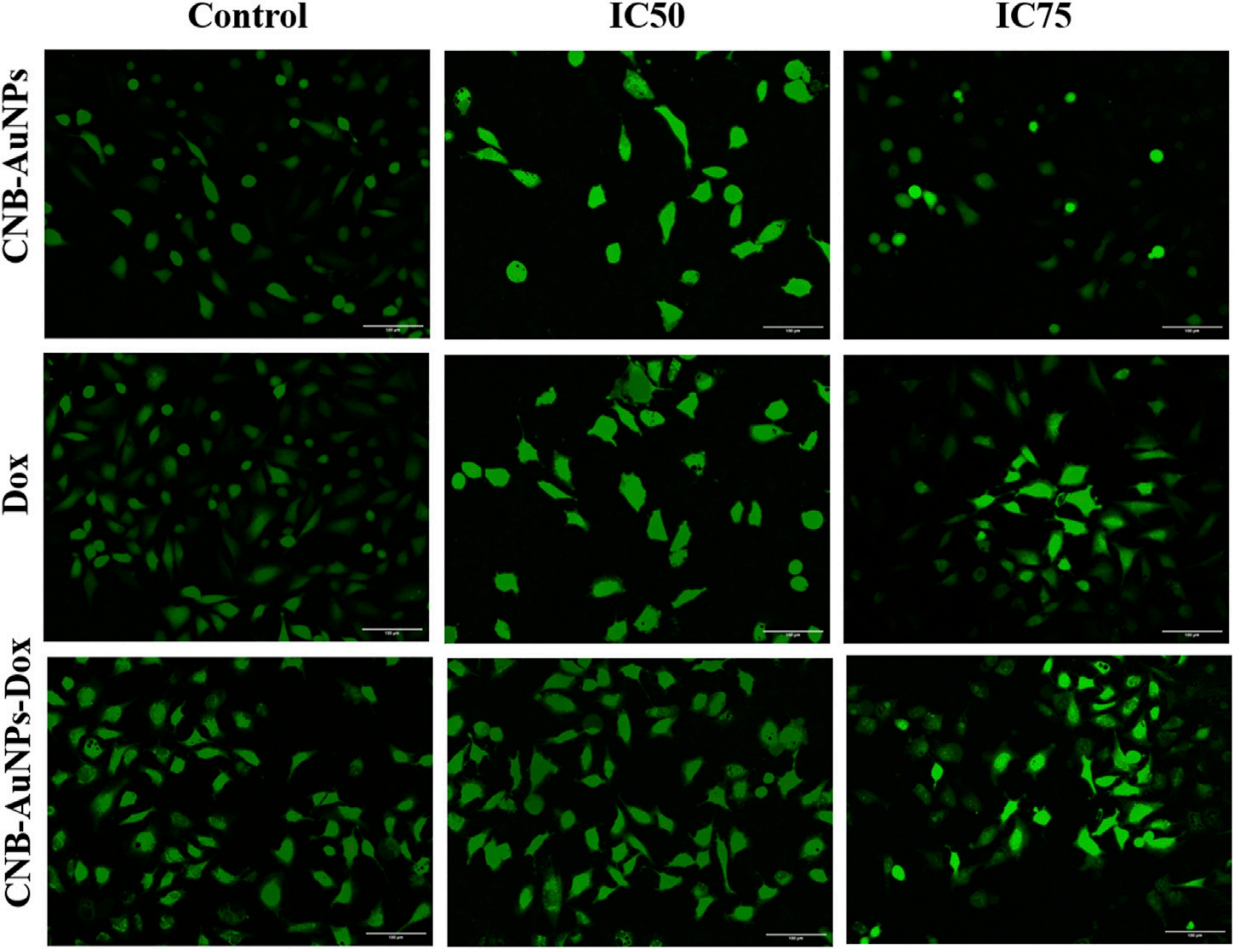
Qualitative evaluation of ROS in H_2_DCFDA stained A549 cells treated with IC50 and IC75 of CNB-AuNPs-Dox, Dox, and CNB-AuNPs for 24 h analyzed by fluorescence microscopy. The images shown are representative of three independent experiments (Scale bar: 100μm; Magnification: ×20).

**FIGURE 10:**
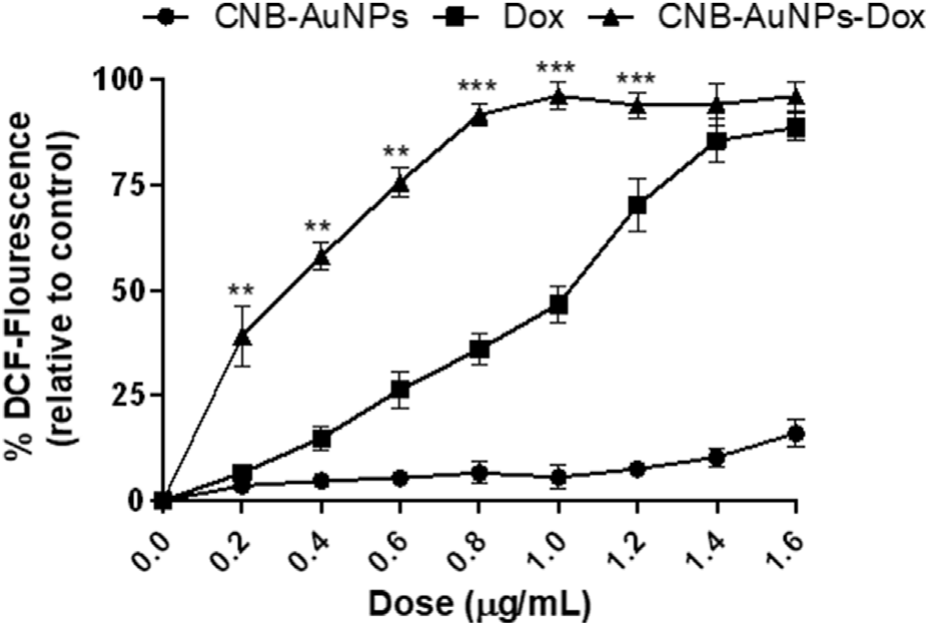
Quantitative determination of ROS in H_2_DCFDA-stained A549 cells treated with various concentrations of CNB-AuNPs-Dox, Dox, and CNB-AuNPs for 24 h. The results presented are the mean ± SD of three independent experiments performed in triplicate. Significance among different dosage groups was determined using two-way ANOVA followed by Tukey’s multiple comparison tests (where *p < 0.05, **p < 0.01, and ***p < 0.001 represent significant differences between means of different treatment groups).

The escalation of intracellular ROS generation has been widely documented as a significant impetus for the initiation of apoptosis. Therefore, to investigate, whether drug-induced apoptosis in A549 cells was due to intracellular ROS generation, H_2_DCFDA staining was done. The intensity of DCF fluorescence is directly proportional to the intracellular level of ROS. Among all the treatment groups, A549 cells showed a dose-dependent increase in DCF fluorescence, whereas insignificant fluorescence was observed in untreated control cells (Figure 9). As the treatment dose was increased from IC_50_ to IC_75_, ROS generation in A549 cells was further increased in all the treatment groups. As observed above, CNB-AuNPs-Dox caused comparable augmentation in ROS generation in A549 cells at lower doses when compared with Dox alone. In an additional experiment, intracellular ROS level was quantified and the result was shown as percent DCF-fluorescence relative to control in A549 cells among all treatment groups. A concentration-dependent augmentation in DCF-fluorescence was observed in A549 cells treated with CNB-AuNPs-Dox as well as Dox (Figure 10). However, CNB-AuNPs-Dox-treatment induced greater ROS production in A549 cells, at similar doses, when compared to Dox alone.

### Activation of caspases

**FIGURE 11:**
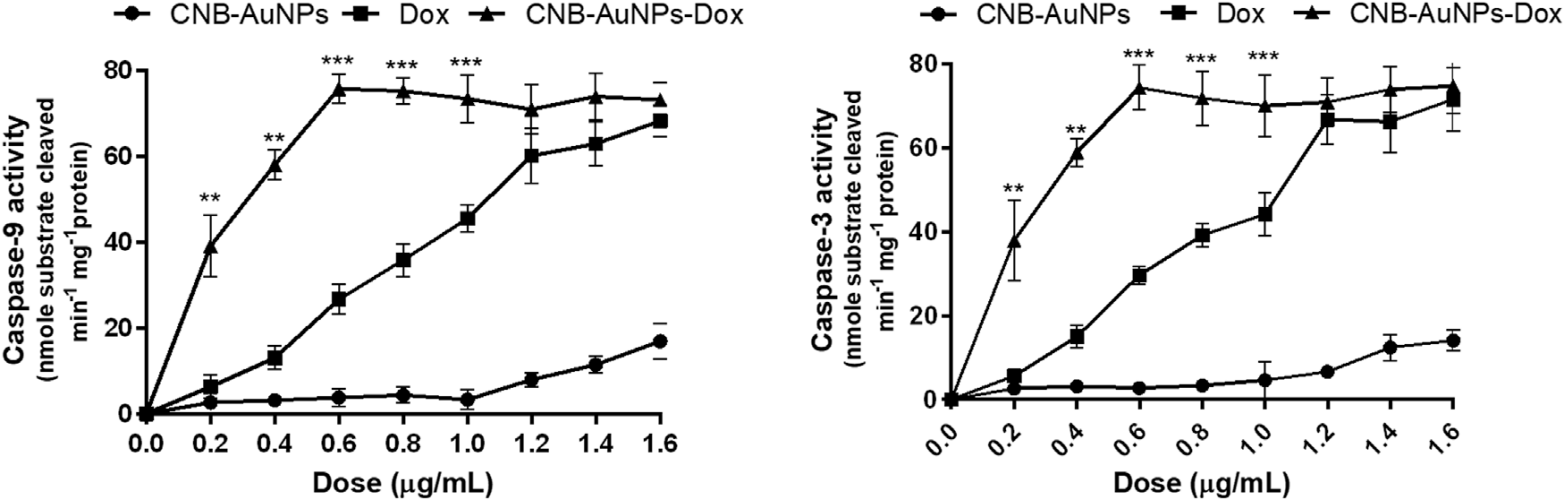
Estimation of Caspase-9, and Caspase-3 activities in A549 cells treated with various concentrations of CNB-AuNPs-Dox, Dox, and CNB-AuNPs for 24 h. The results presented are the mean ± SD of three independent experiments performed in triplicate. Significance among different dosage groups was determined using two-way ANOVA followed by Tukey’s multiple comparison tests (where *p < 0.05, **p < 0.01, and ***p < 0.001 represent significant differences between means of different treatment groups).

As caspase-9 and -3 play a key role in activating the intrinsic pathway, we set out to determine whether the induction of apoptosis in CNB-AuNPs-Dox-treated A549 cells was due to activation of caspases. Thus, caspase-9 and -3 activity were measured in A549 cells treated with varying concentrations of CNB-AuNPs-Dox as well as Dox and CNB-AuNPs. Our results showed a significant augmentation in caspase-3 and -9 activities in A549 cells, treated with CNB-AuNPs-Dox as well as Dox (Figure 11). However, CNB-AuNPs-Dox exerted more pronounced caspase-3 and -9 activities in A549 cells in comparison to Dox alone, at similar doses.

## Discussion

Around 85% of lung cancer incidences are characterized as non-small cell lung cancer (NSCLC). It is the foremost contributor to tumor-related mortality globally, underscoring the necessity for improved and efficacious therapeutic interventions (Rathos et al., 2013). NSCLC exhibits inherent resistance and typically lacks responsiveness to primary chemotherapy. The administration of doxorubicin as a therapeutic intervention for advanced NSCLC yields a mere 30%–50% overall response rate (Rathos et al., 2013). Unfortunately, the acute and cumulative toxicity related to the dose, in conjunction with significant drug resistance, presents a significant challenge to therapeutic outcomes. In this regard, the nanoparticle-based drug delivery method has been shown to be very effective in cancer therapy (Santiago et al., 2017; Singh et al., 2018b). The biocompatibility and lack of toxicity exhibited by AuNPs render them an outstanding contender for drug delivery purposes (Ajnai et al., 2014; Kong et al., 2017). Several researchers have employed chemotherapy as a treatment modality for lung carcinoma utilizing Dox (Amreddy et al., 2015; Lv et al., 2017; Singh, Singh, Sahu, Srivastava and Singh, 2016; Zhang et al., 2015; Zhang et al., 2016). Nevertheless, the utilization of this therapeutic agent in clinical settings is restricted due to its adverse side effects, with cardiotoxicity being the most remarkable. Hence, researchers are endeavoring to design novel delivery mechanisms in order to curtail its adverse reactions and intensify its therapeutic potency. To date, no study has reported the conjugation of Dox with AuNPs, synthesized by using *C. sativa L.* extract. Therefore, we studied the potential of AuNPs to serve as a nanocarrier for Dox.

Biogenically synthesized CNB-AuNPs were characterized using UV-Vis spectroscopy. The confirmation of CNB-AuNPs synthesis was ascertained through the observation of an absorption peak at a wavelength of 524 nm. Our findings are consistent with a previous study in which it was demonstrated that the highest peaks of AuNPs fall within the range of 520–560 nm (Aldalbahi et al., 2020). CNB-AuNPs exhibit a zeta potential value of −20.1 mV, indicating that these nanoparticles possess notable stability owing to the electrostatic repulsive force (Leite et al., 2012). The hydrodynamic diameter of gold nanoparticles was determined to be 45.64 nm through the utilization of the dynamic light scattering (DLS) technique. The identification of the size and morphology of AuNPs was carried out via the utilization of TEM. The observed average size of CNB-AuNPs was determined to be approximately 17.2 nm. After the conjugation reaction, the samples were analyzed by various biophysical techniques to characterize the conjugation of Dox with AuNPs. CNB-AuNPs-Dox were observed to exhibit a pronounced peak at 527 nm upon analysis via UV-Vis spectroscopy. The hydrodynamic diameter of CNB-AuNPs-Dox was determined to be 58.95 nm. This observed increase in size may be attributed to the conjugation of Dox with CNB-AuNPs. The high stability of the nanoemulsion of CNB-AuNPs-Dox is also evidenced by its negative zeta potential of −12.7 mV. The findings of the TEM analysis demonstrated that the CNB-AuNPs-Dox exhibited an average size of 23.5 nm, which was marginally larger in comparison to the CNB-AuNPs. This can be attributed to the coupling of Dox over the surface of CNB-AuNPs. Our findings are consistent with a prior report where Dox has been shown to conjugate with iron oxide nanoparticles (Liu et al., 2016). Another study also reported the conjugation of Dox with zinc nanoparticles (Sharma et al., 2016). In an interesting study, Dox has been shown to conjugate with zinc oxide nanoparticles using glutaraldehyde (Ruenraroengsak et al., 2019).

The results of the cytotoxicity assay showed that CNB-AuNPs-Dox as well as DOX were effective in reducing the proliferation of A549 cells; however, the effectiveness of CNB-AuNPs-Dox was found to be significantly increased at lower doses when compared to Dox. The enhanced activity of CNB-AuNPs-Dox can most plausibly be attributed to the high drug loading capacity of the gold-nanocarrier and effective drug delivery. This could potentially lead to a significant increase in the intracellular concentration of Dox, ultimately resulting in an enhanced reduction in cancer cell proliferation. Our findings are consistent with a prior investigation, in which Dox, when conjugated to AuNPs utilizing an acid-labile linker, exhibited heightened cytotoxicity against the multidrug-resistant MCF-7/ADR breast cancer cell line. This indicates that the AuNPs-bound drug is taken up more readily and subsequently released within the cell responsively, resulting in a partial reversal of multidrug resistance (Wang et al., 2011). In an additional *in vitro* investigation, it was observed that conjugates of oxaliplatin with PEG-coated/carboxylate-capped AuNPs exhibit a greater degree of cytotoxicity as compared to free oxaliplatin in HCT15, HT29, and RKO colon cancer cell lines (Brown et al., 2010). 5-fluorouracil (5-FU), conjugated with glutathione-caped AuNPs, exhibited an enhanced anticancer effect in colorectal cancer cells when compared to 5-FU alone (Safwat et al., 2016). The cytotoxicity of Capecitabine, cisplatin, or doxorubicin conjugated to L-aspartate-stabilized AuNPs was found to be higher towards hepatocellular carcinoma cells than that of free capecitabine, free cisplatin or doxorubicin (Tomuleasa et al., 2012). In another *in vitro* investigation, the conjugation of etoposide to hydroxy propyl methylcellulose/polyvinyl alcohol-functionalized AuNPs exhibited enhanced cytotoxicity effects on the NCI-H69 cell line in comparison to free etoposide (Ali et al., 2020). Methotrexate (MTX), when conjugated with AuNPs, exhibited increased cytotoxicity against a variety of tumor cell lines in comparison to free MTX. The accumulation of MTX in tumor cells was observed to occur at a more rapid pace and to a greater extent when conjugated with AuNPs (Chen et al., 2007). These studies support the high drug loading efficiency of AuNPs which can be utilized to formulize novel drug nanoformulations. Although we have not studied how the Dox is delivered inside the cell by CNB-AuNPs, the previous studies have established that the energy-dependent endocytic pathways are important for the internalization of nanoparticles inside the cell (Hong et al., 2009; Mailander and Landfester, 2009).

Furthermore, cytomorphological analysis, performed by phase contrast microscopy, showed that the shapes of A549 cells were significantly changed in all the treatment groups at respective IC_50_ and IC_75_, and were characterized by shrinking and detachment. Likewise, DAPI staining in A549 cells, at respective IC_50_ and IC_75_, showed chromatin condensation with bright blue nuclei, indicating initiation of apoptosis. Furthermore, CNB-AuNPs-Dox exerted similar alterations in A549 cells at lower doses in comparison to Dox alone. As shown previously, the loss of Δψ_m_ is an early hallmark of apoptosis (Khan et al., 2018). Interestingly, A549 cells, at respective IC_50_ and IC_75_, showed a gradual decrease. CNB-AuNPs-Dox reduced the Δψ_m_ in A549 cells at lower concentrations in comparison to DOX signifying effective intracellular delivery of the drug.

The onset of elevated basal oxidative stress can be attributed to the overproduction of ROS in cancer cells. The state of oxidative stress that cancer cells experience renders them susceptible to therapeutic agents that could enhance ROS levels. Hence, pro-oxidants are increasingly being viewed as promising candidates to selectively target tumor cells (Martin-Cordero et al., 2012). Qualitative examination depicted augmentation in the ROS level in A549 cells in all the treatment groups at respective IC_50_ and IC_75_. CNB-AuNPs-Dox induced a comparable amount of ROS generation in A549 cells at lower concentrations as compared to Dox alone. Additionally, quantitative examination of ROS showed that CNB-AuNPs-Dox, as well as Dox, were efficient in increasing ROS generation in A549 cells; nevertheless, the effectiveness of CNB-AuNPs-Dox was more reflective at lower doses when compared to Dox alone.

Caspases are produced in an inert proenzyme state and their activation during the process of apoptosis leads to cleavage at distinct sites (Enari et al., 1998; Thornberry et al., 1997). The caspases-8 and -9, acting as initiators, undergo self-catalytic activation, while the caspase-3, responsible for execution, is activated by the aforementioned initiator caspases. Caspase-3 is a critical mediator of programmed cell death and is accountable for the proteolytic cleavage of numerous cellular proteins (Enari et al., 1998). Results of the study revealed that CNB-AuNPs-Dox as well as Dox caused significant caspase-3 and -9 activation in A549 cells; though, the effect of CNB-AuNPs-Dox was more pronounced at lower doses when compared to Dox.

## Conclusion

In the present investigation, we have demonstrated the synthesis of gold nanoparticles through a green chemistry methodology that is simple, effective, cost-efficient, and environmentally sustainable. Several physicochemical methods were used to systematically characterize the nanoparticles. Later, doxorubicin-gold nanoconjugates were shown to exert substantial inhibition of lung cancer cell proliferation in comparison to free doxorubicin. In sum, our results demonstrated the efficacy of a biogenic gold nanoparticle-based drug-delivery system against lung cancer cells which could be tested in pre-clinical and clinical settings in the near future for the development of an alternative cost-effective treatment approach for cancer therapy.

## Data Availability

The original contributions presented in the study are included in the article/Supplementary Material, further inquiries can be directed to the corresponding authors.
