# Supplementary figures and images for: Biogenically synthesized gold nanocarrier ameliorated antiproliferative and apoptotic efficacy of doxorubicin against lung cancer
Source: Front Pharmacol. 2024 Oct 29;15:1438237. doi: 10.3389/fphar.2024.1438237 (PMC11555439; doi:10.3389/fphar.2024.1438237)

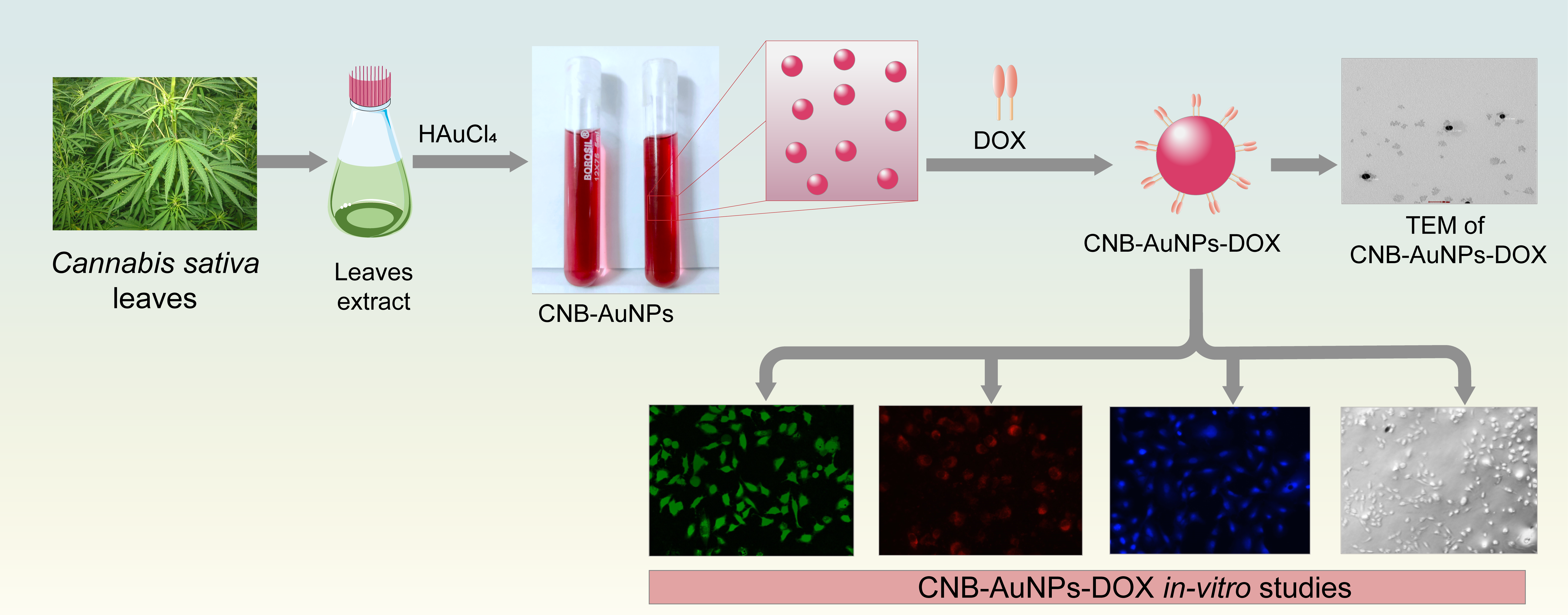

Supplement: Supplementary file 1 [file Image1.TIF]
